# Insights into the Middle Eastern paternal genetic pool in Tunisia: high prevalence of T-M70 haplogroup in an Arab population

**DOI:** 10.1038/s41598-021-95144-x

**Published:** 2021-08-03

**Authors:** Sarra Elkamel, Sofia L. Marques, Luis Alvarez, Veronica Gomes, Sami Boussetta, Soufia Mourali-Chebil, Houssein Khodjet-El-Khil, Lotfi Cherni, Amel Benammar-Elgaaied, Maria J. Prata

**Affiliations:** 1 Laboratory of Genetics, Immunology and Human Pathology, LR 05ES05, Faculté de Sciences de Tunis, Université de Tunis El Manar, 2092 Tunis, Tunisia; 2 Institute for Research and Innovation in Health Sciences (i3S), University of Porto, Porto, Portugal; 3 Institute of Molecular Pathology and Immunology, University of Porto, Porto, Portugal; 4 Department of Biomedical Sciences, College of Health Sciences, QU Health, Qatar University, Doha, Qatar; 5 High Institute of Biotechnology, University of Monastir, 5000 Monastir, Tunisia; 6 Faculty of Sciences, University of Porto, Porto, Portugal

**Keywords:** Genetics, Population genetics

## Abstract

To obtain refreshed insights into the paternal lineages of Tunisian populations, Y-chromosome diversity was assessed in two populations belonging to an Arab genealogical lineage, Kairouan and Wesletia, as well as in four Tunisian Andalusian populations, Testour, Slouguia, Qalaat-El-Andalous and El Alia. The Arabs from Kairouan revealed 73.47% of E-M81 and close affinities with Berber groups, indicating they are likely arabized Berbers, clearly differentiated from the Arabs from Wesletia, who harbored the highest frequency (71.8%) of the Middle Eastern component ever observed in North Africa. In the Tunisian Andalusians, the North African component largely prevailed, followed by the Middle Eastern contribution. Global comparative analysis highlighted the heterogeneity of Tunisian populations, among which, as a whole, dominated a set of lineages ascribed to be of autochthonous Berber origin (71.67%), beside a component of essentially Middle Eastern extraction (18.35%), and signatures of Sub-Saharan (5.2%), European (3.45%) and Asiatic (1.33%) contributions. The remarkable frequency of T-M70 in Wesletia (17.4%) prompted to refine its phylogeographic analysis, allowing to confirm its Middle Eastern origin, though signs of local evolution in Northern Africa were also detected. Evidence was clear on the ancient introduction of T lineages into the region, probably since Neolithic times associated to spread of agriculture.

## Introduction

North Africa experienced a rich and complex population history since ancient times, which is understandable given its strategic position across the southern part of the Mediterranean Sea. Tunisia, the northernmost country in Africa, holds a privileged geographic position representing a crossroad between Africa, Europe and the Middle East. According to archaeological and historical data, the settlement of human populations coming from the east has occurred in Tunisia in different prehistoric and historical migration waves. During the Neolithic period, the Capsian culture that flourished in the Maghreb, between 10,000 and 5000 years ago, left remarkable sites in nowadays Southern Tunisia, as attested by the archaeological traces in Gafsa^1–3^. The emergence of agriculture and animal domestication was splendidly captured by the cave paintings and rock engravings in the mountains of Jebel Ousselat^4,5^. In historical times, Tunisia was primarily inhabited by autochthonous Berber populations that faced the arrival of successive waves of invaders, most of them coming from the Middle East: i) Phoenicians, originally from Lebanon, who founded Carthage in the twelfth century BC; later ii) the Arab Muslims who conquered the region in the seventh century that would lead to the foundation of Kairouan, the first Islamic city in Northwest Africa; and later on iii) the Bedouins in consequence of the invasion of Banu Hilal tribes coming from the Arabian Peninsula in the eleventh century. This last event would instigate the Arabization and Islamization of many Berber populations. More recently, in the sixteenth century, when Tunisia was incorporated into the Ottoman Empire, a substantial migration of Turkish people coming from other eastern Ottoman territories took place. In the beginning of the seventeenth century a new influence to the Tunisian population scenario took place with the entry of the “Andalusians”, the name given to the Muslims fleeing from Spain after the decline of the Muslim rule of the Iberian Peninsula, initiated with the conquest of al-Andalus in 711 and lasting till the fall of Granada in 1492^6^. The following decades were turbulent times for the Muslims communities in Spain, whose persecution would culminate in the years 1609 to 1614 with the final expulsion of the Moriscos (designation given to Muslim converted to Christianity) from the whole nation, a massive event during which thousands of people were driven from their homes, the majority to North Africa^7^.

Several studies have already been undertaken to understand the genetic structure of North African human populations and discern the origin and timing of the migrations that modeled current-day populations. In this work we sought to finely dissect variability of the paternal genetic pool in Tunisia in order to infer the past population interactions, especially addressing the paternal Middle Eastern inputs.

Previous works with Y-chromosome markers^8–22^ have shown that in North Africa the most widespread and common Y lineages are the E- M81, which reaches an average frequency of 45% across the region^15,20,21^ and is thought to have a very recent origin probably in Northwest Africa^19^, and J-M267, accounting for around 30% of North Africans and assumed to have spread out of the Arabia Peninsula into North Africa, as is shown by, for instance, its east–west decreasing prevalence^11,14,15,20,21,23^. There is wide agreement about the sources of J-M267 in North Africa, which imply multiple and temporarily different expansions of Middle-Eastern and Arabic populations through the Mediterranean. In fact, although this haplogroup is considered to be one of the signatures of the spread of Islam from the Arabian Peninsula^15,24–30^, it also retains clues on a much earlier expansion during Neolithic times as part of the previously mentioned Capsian cultural complex that was introduced in North Africa along with agriculture^9,12,15,29,31^.

Another haplogroup consistently associated to the package of lineages accounting for the rapid expansion of Neolithic farmers from the Middle East is J-M172^25,28,32^, whose recent spread in the Mediterranean is believed to have been facilitated by the maritime trading culture of the Phoenicians^15^. Besides, G-M201 and T-M184 haplogroups, also supposed to have originated in the Middle East, were occasionally detected in some North African populations and their distribution also suggests their involvement in the Neolithization of North Africa^33,34^.

In this context, we sought to refine the knowledge on the extent of the paternal Middle Eastern contribution in Tunisia. Because previous studies on Tunisian populations have mainly included cosmopolitan citizens, Berbers or “Andalusian” groups, we set as goal to enrich the catalogue of Tunisia populations by studding Tunisians claimed to have stronger Middle Eastern contribution. To that end, we assessed Y-STRs and Y-SNPs diversity in samples from Kairouan and Wesletia known to belong to Arab genealogical lineages. Kairouan, founded in the 7th CE, was indeed the first Islamic Arab city in North Africa that would become the capital of Ifriqiya (roughly equivalent to modern Tunisia) for five centuries (7th to 11th CE). It houses the Great Mosque, one of the most important monuments in Arabo-Islamic architecture that functioned as a centre of Islamic thought, attracting scholars from all over the Islamic World^35,36^. Contrarily, Wesletia is a rural city surrounded in the east by the mountains of Jebel Ousselat, which preserve the richest rock art in Tunisia since the Neolithic period^4,5^. In previous works, we have already characterized both populations for 15 autosomal STRs loci^37^ and mtDNA HVS-I and HVS-II regions^38^, which disclosed the clustering of North African and Middle Eastern populations^37^ and further showed that in the Middle Eastern maternal genetic pool of Tunisian populations prevailed an ancient substrate suggesting that migration waves during Paleolithic and Neolithic periods must have been more important contributors to that component than the much more recent Islamic expansion that brought the massive Bedouin dispersal in the 11th CE^38^.

In the present work we aimed at discerning the paternal Middle Eastern lineages in present-day populations of recent Arab ancestry from Kairouan and Wesletia and compare the findings with those obtained in four previously studied Tunisian Andalusian populations (Testour, Slouguia, Qalaat-El-Andalous and El Alia)^39,40^. In addition, a comprehensive phylogenetic analysis of the Middle Eastern Y-chromosome haplogroup T was performed after recruiting available data from the literature to further elucidate the extent of gene flow from the Middle East into North African populations.

## Results

### Distribution of Y chromosomal haplogroups in Tunisian populations

**Figure. 1:**
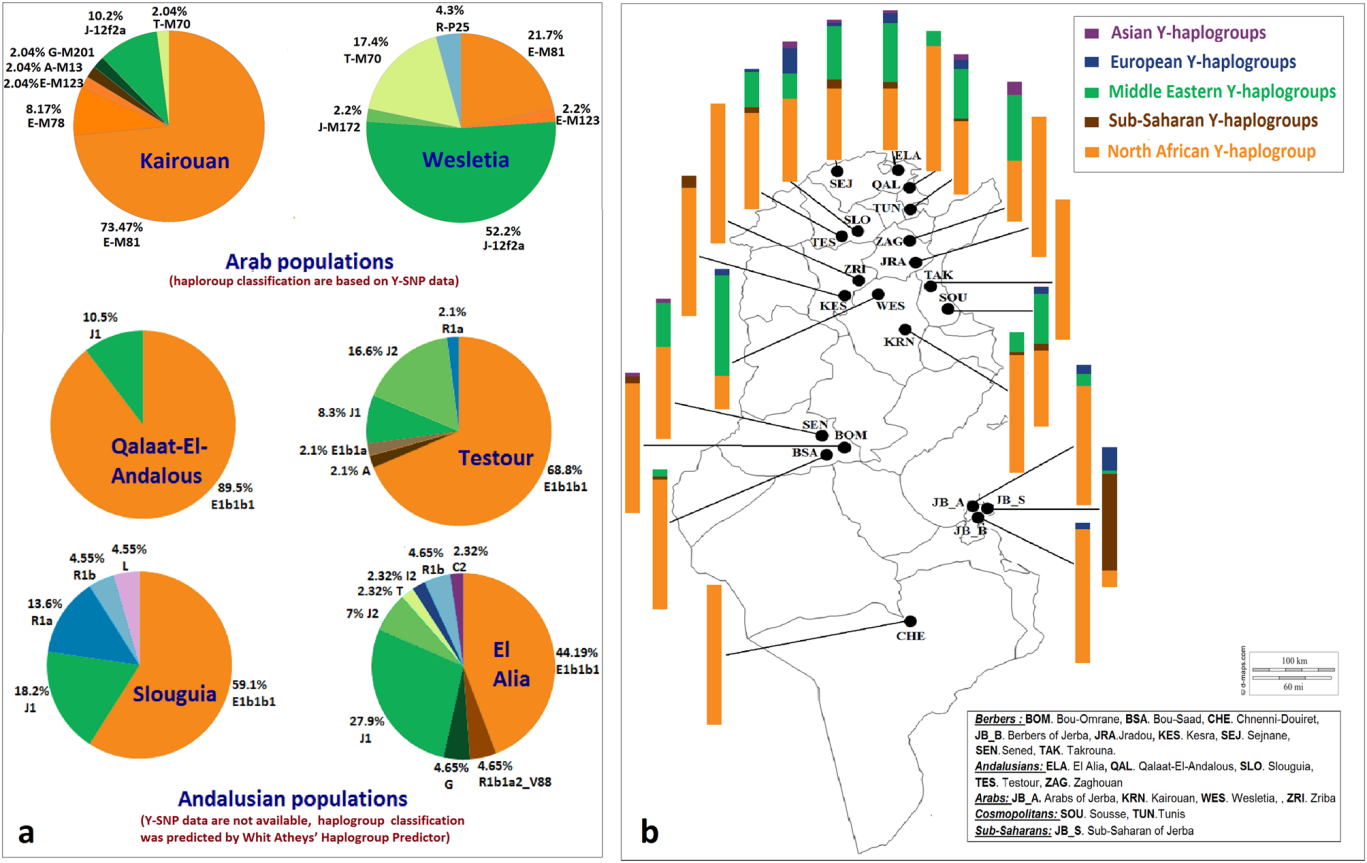
Distribution of Y-chromosomal haplogroups in Arab and Andalusian populations (**a**) and in 21 Tunisian populations (**b**) (details of Y-Chromosome haplogroups frequencies in Supporting Information Table S2).

In the present work, we analyzed 23 Y-STRs and 29 Y-SNPs in Tunisian Arabs from Kairouan and Wesletia (haplotypes and haplogroups in Supporting Information Table S1). To investigate the contribution of male Middle Eastern lineages in Tunisia, we centred attention on the distribution of Y chromosomal haplogroups in the two studied Arab populations, in four Tunisian Andalusian populations (Fig. 1a), and other Tunisian populations (Fig. 1b) previously characterized (Supporting Information Table S2).

Unexpectedly, a strong differentiation was found between the samples from Kairouan and Wesletia. In the Arabs from Kairouan clearly prevailed a genetic male background of Berber ancestry, witnessed by the very high frequency of North African sub-clades (83.68%), contrasting with the much lower proportion of Middle Eastern (14.28%) or the residual value of Sub-Saharan lineages (2.04%). Contrarily, in the Arabs from Wesletia, haplogroups typically found in Middle Eastern populations were very well represented, summing up a combined frequency of 71.8%, whereas the North African substrate was detected at the moderate frequency of 23.9% and the European only attained the frequency of 4.3% (Fig. 1a).

Among the Tunisian Andalusian populations, stand out Qalaat-El-Andalous by the high frequency of the North African lineages (89.5%), and El Alia by exhibiting the highest proportion of the Middle Eastern ones (41.87%). In Testour and Slouguia, the most frequent haplogroups are North African (68.8% and 59.1%, respectively), the Middle Eastern assumes intermediate values (24.9% and 18.2%, respectively), while the European, Sub-Saharan and Asian are present in both populations with low frequencies (Fig. 1a).Overall, the paternal pool in Tunisia is clearly dominated by E-M35 and J-M267, two lineages that capture the trace of North African and Middle Eastern ancestry, respectively (Fig. 1b).

The distribution of the E-M35 sub-clades (including mainly E-M81, bust also E-M183, E-M78, E-M123, E-V13, E-V22, E-V65, E-M215) is marked by the high average frequency (around 72%), although showing remarkable disparities between populations (Fig. 1b; Supporting Information Table S2).

Regarding the Middle Eastern contribution, J-M267 haplogroup is present at the average frequency of 15% in Tunisian populations, but as well shows large inter-population variability, ranging from 52.2% in Arabs from Wesletia, 43.75% in Tunisian Andalusians from Zaghouan to 0% in Sub-Saharans from Jerba and Berber populations (Chenini-Douiret, Jradou, Takrouna, Berbers of Jerba, Bou-Omrane and Kesra). In turn, J-M172 is comparatively less frequent, varying from 16.6% in Andalusians from Testour, 8.2% in Cosmopolitans from Sousse, to 0% in 13 out of 21 Tunisian populations. Other Middle Eastern subclades such as G-M201, T-M184 and T-M70 were residually observed, except T-M70 in the Arabs from Wesletia detected at the remarkable frequency of 17.4%.

As for the Sub-Saharan influence (inferred through haplogroups such as A-M13, A-M91, B-M218, DE-YAP, E-M96, E-M132, E-V38, and R-V88), it is very strong in the sub-Saharans from Jerba, among whom E-V38 and R-V88 represent 33.3% and 35.7%, respectively, of the male lineages.

The proxies of the European component here identified were R-M207, R-M198, R-P25, I-M253 and I-M438 sub-clades, each one occasionally detected in some populations; their combined average frequency did not exceed around 3%. Sporadic occurrences of a few Asian haplogroups like C-M217, L-M20, F-M89 and Q-M346 were also found with the average frequency of ~ 1%.

In sum, the Tunisian paternal pool is mainly dominated by a North African component (71.67%), followed by a Middle Eastern contribution (18.35%) and, at much lesser extent, by lineages of Sub-Saharan (5.2%), European (3.45%) and Asiatic (1.33%) origin.

### Y-STR genetic diversity in Tunisia

Considering the 23 Y-STR analyzed in the two Tunisian Arab samples, we found 45 (91.83%) and 39 (84.78%) different haplotypes in Kairouan and Wesletia, respectively. Both populations display high haplotype diversities (0.996 ± 0.004 in Kairouan and 0.992 ± 0.006 in Wesletia) and the mean number of pairwise differences in Wesletia is higher (12.639) than in Kairouan (9.929). Diminishing the resolution to 17 Y-STR (Table 1), the number of different haplotypes was obviously more reduced (85.71% in Kairouan and 69.46% in Wesletia).

**Table 1:** Y-chromosome genetic diversity parameters in Tunisian populations.

Ethnic group	Population	*N*	17 Y-STR			12 Y-STR		
			*K*	*HD*	*MNPD*	*K*	*H* ± *SD*	*MNPD*
*Arabs*	Kairouan	49	42	0.993 ± 0.005	6.850 ± 3.280	31	0.953 ± 0.018	4.455 ± 2.234
	Wesletia	46	32	0.973 ± 0.012	8.713 ± 4.096	25	0.950 ± 0.016	5.989 ± 2.908
	Zriba	31	–	–	–	5	0.569 ± 0.079	1.165 ± 0.771
*Andalusians*	Zaghouan	32	29	0.994 ± 0.009	7.590 ± 3.636	24	0.977 ± 0.013	5.340 ± 2.645
	Qalaat-El-Andalous	19	–	–	–	5	0.386 ± 0.138	1.982 ± 1.170
	El Alia	43	–	–	–	32	0.979 ± 0.011	6.913 ± 3.315
	Testour	48	–	–	–	37	0.984 ± 0.009	6.453 ± 3.108
	Slouguia	22	–	–	–	17	0.969 ± 0.024	6.372 ± 3.139
*Berbers*	Sejenane	47	36	0.969 ± 0.017	8.574 ± 4.034	29	0.942 ± 0.021	5.709 ± 2.784
	Takrouna	19	12	0.900 ± 0.058	4.105 ± 2.139	9	0.777 ± 0.095	2.426 ± 1.376
	Chenini-Douiret	27	23	0.988 ± 0.013	5.931 ± 2.921	17	0.960 ± 0.020	4.415 ± 2.248
	Jradou	32	14	0.840 ± 0.055	2.362 ± 1.322	5	0.671 ± 0.061	1.411 ± 0.886
	Sened	35	25	0.976 ± 0.013	6.744 ± 3.256	18	0.890 ± 0.043	4.142 ± 2.111
	Bou-Omrane	40	11	0.809 ± 0.051	3.179 ± 1.679	7	0.715 ± 0.048	1.993 ± 1.148
	Bou-Saad	40	18	0.946 ± 0.015	3.960 ± 2.024	8	0.639 ± 0.075	1.930 ± 1.120
	Kesra	23	–	–	–	14	0.905 ± 0.051	3.173 ± 1.703
*Cosmopolitans*	Tunis	33	30	0.992 ± 0.010	9.034 ± 4.267	27	0.981 ± 0.014	6.060 ± 2.960
	Sousse	218	156	0.991 ± 0.002	9.254 ± 4.269	125	0.975 ± 0.004	6.346 ± 3.020

N: Sample size, K: Number of different haplotypes, HD: Haplotype diversity, MNPD: Mean Number of Pairwise Differences.

The diversity parameters in other Tunisian populations previously studied (Arabs, Berbers, Tunisian Andalusians and Cosmopolitans) are also shown in Table 1. The genetic diversity indices inferred from 12 Y-STR (shared by all compared populations) show that in general non-Berber Tunisian populations harbour high level of haplotype diversity, with values ranging from 0.984 ± 0.009 to 0.950 ± 0.016, either in Cosmopolitans, Tunisian Andalusians (except Qalaat-El-Andalous, 0.386 ± 0.138) or Arabic populations (except Zriba, 0.569 ± 0.079). Berber populations typically show lower haplotype diversities, varying from 0.960 ± 0.020 in Chenini-Douiret to 0.639 ± 0.075 in Bou-Saad. MNPD values are usually high except for Arabs from Zriba, Andalusians from Qalaat-El-Andalous and Berbers from Jradou, Bou-Omrane and Bou-Saad.

### Paternal genetic structure in Tunisian populations

#### Genetic distances

Pairwise *R*_*ST*_ genetic distances (based on 12 Y-STR) and corresponding *P*-values between 18 Tunisian populations are given in Supporting Information Table S3. After applying the Bonferroni correction (*P* = 0.00277), the Arabic populations (Wesletia, Kairouan and Zriba) differed significantly from each other as well as from most of the remaining Tunisian populations. Non-significant differentiations emerged between i) Wesletia and Tunisian Andalusians from Zaghouan, ii) Wesletia and Cosmopolitans from Tunis on the one hand and Kairouan and Cosmopolitans of Sousse on the other, or iii) Kairouan and Berbers from Takrouna as well as Zriba and Berbers from Takrouna. Remarkably, most of the genetic distances between Cosmopolitans and Tunisian Andalusian populations were statistically non-significant.

The *R*_*ST*_ genetic distances were used to generate a multidimensional scaling (MDS) plot outlining the relationships between Tunisian populations (Supporting Information Figure S1), which discloses a vague ethnic-based structure, in the sense that most of the Berber populations (except Sejnane) are grouped on the negative side of one the axes, while Cosmopolitans and Tunisian Andalusians are positioned on the positive side. Also of note the relative scattering of Berbers, thereby confirming their previously described sharp genetic heterogeneity^10,13,39,41,42^. The so-called Arab populations escape the structure before mentioned once Zriba and Kairouan are closer to the Berbers whereas Wesletia (with the largest Middle Eastern component) is near the Tunisian Andalusians.

#### AMOVA and SAMOVA

To further assess the genetic structure of Tunisian populations, we implemented hierarchical AMOVA pooling populations according to the broad ethnical classification into four groups: Berbers, Arabs, Tunisian Andalusians and Cosmopolitans. Results revealed no statistically significant variance among groups (Table 2), sustaining that such common perception of ethnicity is irrelevant for the male genetic structure among Tunisian populations, as already evidenced before^12^. Yet, the variance among populations within groups was highly significant, reflecting the complexity of other factors that account for the pattern of genetic differentiation between Tunisian population^37,43,44^.

**Table 2:** AMOVA: Arabs versus Berbers versus Andalusians versus Cosmopolitans.

Source of variation	Variation (%)	*P* value
Among groups	0.87	0.15445 ± 0.01092
Among populations within groups	12.56	0.00000 ± 0.00000*
Within populations	86.56	0.00000 ± 0.00000*

Arabs: Kairouan, Wesletia, Zriba. Berbers: Sejenane, Takrouna, Chenini-Douiret, Jradou, Sened, Bou Omrane, Bou-Saad, Kesra. Andalusians: Zaghouan, Qalaat-El-Andalous, El Alia, Testour, Slouguia. Cosmopolitans: Tunis, Sousse.

*P value < 0.05.

Then, to assess the influence of geography, we conducted SAMOVA taking into account the geographic location of the populations. Out of the different runs performed from K = 2 to K = 7 groups without a priori classification, the maximum significant value of variation among groups was obtained when 5 groups were considered and only accounted for 15.43% of total variation (Table 3). Overall, the SAMOVA results indicate that the apportionment of Y-STR diversity among Tunisian groups is scarcely influenced by geography, a factor which otherwise exerts the pale role without a clear orientation pattern.

**Table 3:** SAMOVA results for different population partitions.

K	Variation among groups (%)	*P* value	Groups
2	10.5	0.0029*	(Zriba, Bou-Omrane, Bou-Saad); (Kairouan, Wesletia, Sejnane, Takrouna, Chenini-Douiret, Jradou, Sened, Kesra, Zaghouan, Testour, El Alia, Slouguia, Qalaat-El-Andalous, Tunis, Sousse)
3	11.39	0.0029*	(Zriba, Bou-Omrane, Bou-Saad); (Jradou); (Kairouan, Wesletia, Sejnane, Takrouna, Chenini-Douiret, Sened, Kesra, Zaghouan, Testour, El Alia, Slouguia, Qalaat-El-Andalous, Tunis, Sousse)
4	14.14	0.0166*	(Qalaat-El-Andalous); (Wesletia); (Slouguia); (Kairouan, Sejnane, Takrouna, Chenini-Douiret, Jradou, Sened, Zriba, Bou-Omrane, Bou-Saad, Kesra, Zaghouan, Testour, El Alia,Tunis, Sousse)
**5**	**15.43**	0.0000*	**(Takrouna); (Jradou); (Qalaat-El-Andalous); (Zriba, Bou-Omrane, Bou-Saad); (Kairouan, Wesletia, Sejnane, Chenini-Douiret, Sened, Kesra, Zaghouan, Testour, El Alia, Slouguia, Tunis, Sousse)**
6	15.32	0.0000*	(Chenini-Douiret); (Kesra); (Qalaat-El-Andalous); (Takrouna, Jradou); (Bou-Omrane, Bou-Saad, Zriba); (Kairouan, Wesletia, Sejnane, Sened, Zaghouan, Testour, El Alia, Slouguia, Tunis, Sousse)
7	14.87	0.0000*	(Wesletia); (Chenini-Douiret); (Qalaat-El-Andalous); (Bou-Omrane, Bou-Saad); (Zriba, Kesra); (Takrouna, Jradou); (Kairouan, Sejnane, Sened, Zaghouan, Testour, El Alia, Slouguia, Tunis, Sousse)

K: Number of groups; In bold: group with the highest F_CT_ value.

*P value < 0.05.

### Genetic relationships between Tunisian and worldwide populations

**Figure. 2:**
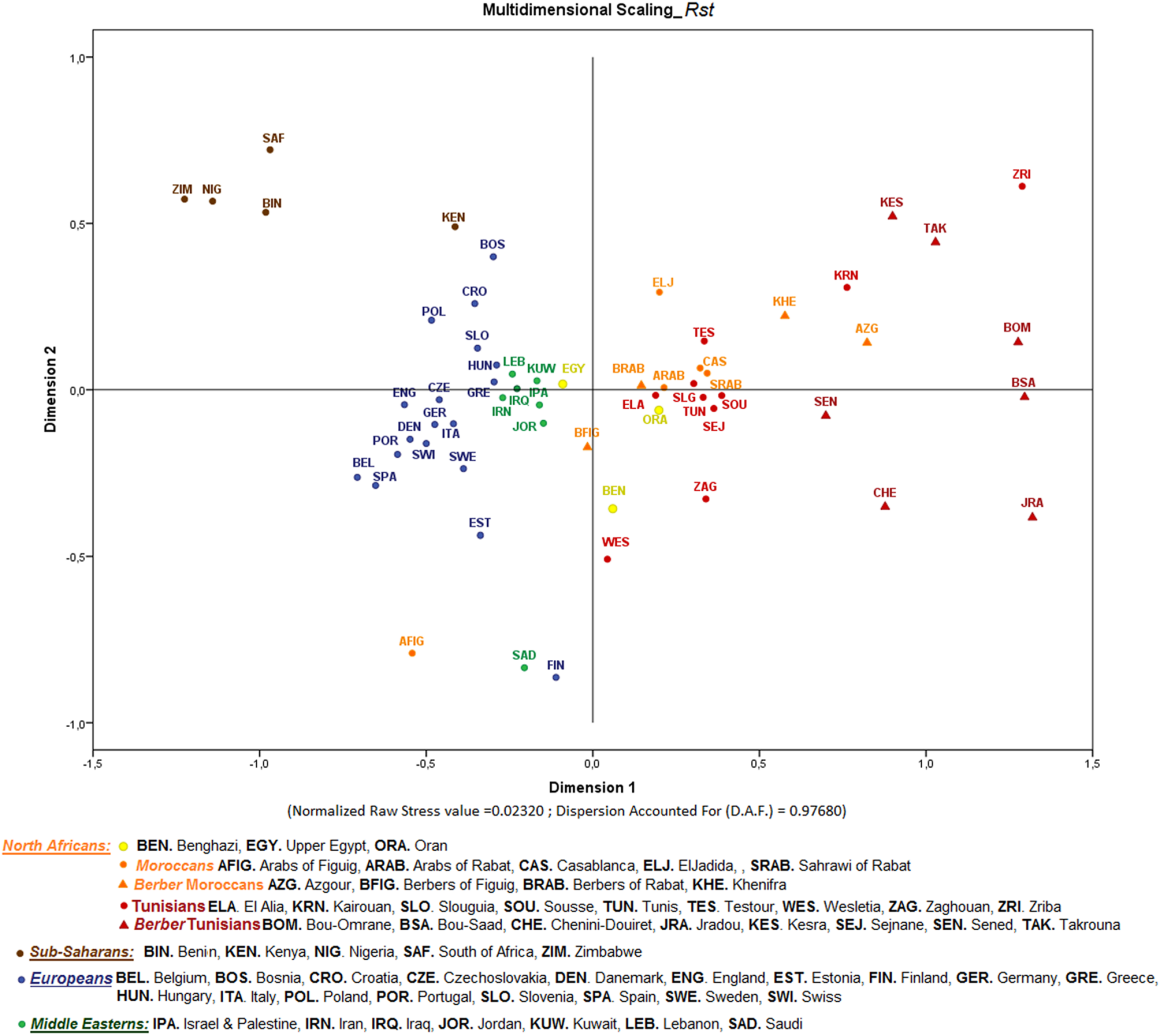
Multidimensional scaling plot based on *R*_*ST*_ genetic distances between pairs of 59 worldwide populations (references of populations in Supporting Information Table S4).

To explore how the two studied Arab populations (Kairouan and Wesletia) were integrated in the diversity context of other populations not only from Tunisia but also from North Africa, Sub-Sahara, Europe and the Middle East, we recruited the available population data (Supporting Information Table S4) ending up with a total 59 populations (15 Tunisians, 12 North Africans, 18 Europeans, 5 Sub-Saharan and 7 from the Middle East). Then, using a resolution of 12 Y-STR loci, those typed in all populations, we performed MDS based on pairwise *R*_*ST*_ genetic distances (Fig. 2) that showed a sharp geographical clustering separating Sub-Saharan, European, and Middle Eastern populations in the negative part of dimension 1 from most of North African populations, including Tunisian ones, in the positive part of that dimension. The proximity of Sub-Saharan populations closer to European than to North African ones is in accordance with previous studies^14,22,45,46^ and the outsider positions of the Kenyans and the Finnish just reflect the peculiarities of these two populations, since the Kenyans considered were Maasai, among whom a considerable degree of endogamy has been reported and the Finnish are known to have passed through a recent bottleneck^46^.

Not devaluating the overrepresentation of Tunisians in this analysis, is of note the diversity of Tunisian populations: the Tunisian Berbers are very dispersed, denoting well their heterogeneity; the Cosmopolitans and Tunisian Andalusians are more central in the plot close to the non-Tunisian North Africans; and the two studied Arab populations are rather apart from each other, with Kairouan near Berber populations and Wesletia showing more affinities with Middle Eastern ones. Within the North African populations, the Egyptian reveals the closest similarities with Middle Eastern ones.

### Phylogeographic analysis for the Y-chromosome haplogroup T in worldwide populations

T-M184 haplogroup, thought to have had origin and initial expansion in the Middle East^34,47^, is globally a rare but geographically widespread haplogroup. It appears at high/intermediate frequencies in populations from the Middle East and East Africa^27,28,47–52^ being fairly rare in Europe, Asia and America^53–58^. Up to now in North Africa, T lineages were sporadically found in most populations, peaking, however, in the eastern ones such as Egyptian (6.7%) and Libyan (2.28%)^30,34^. In this context, it was rather unexpected to have found out that 17.4% of the Tunisian Arabs from Wesletia belonged to sub-haplogroup T-M70.

**Figure. 3:**
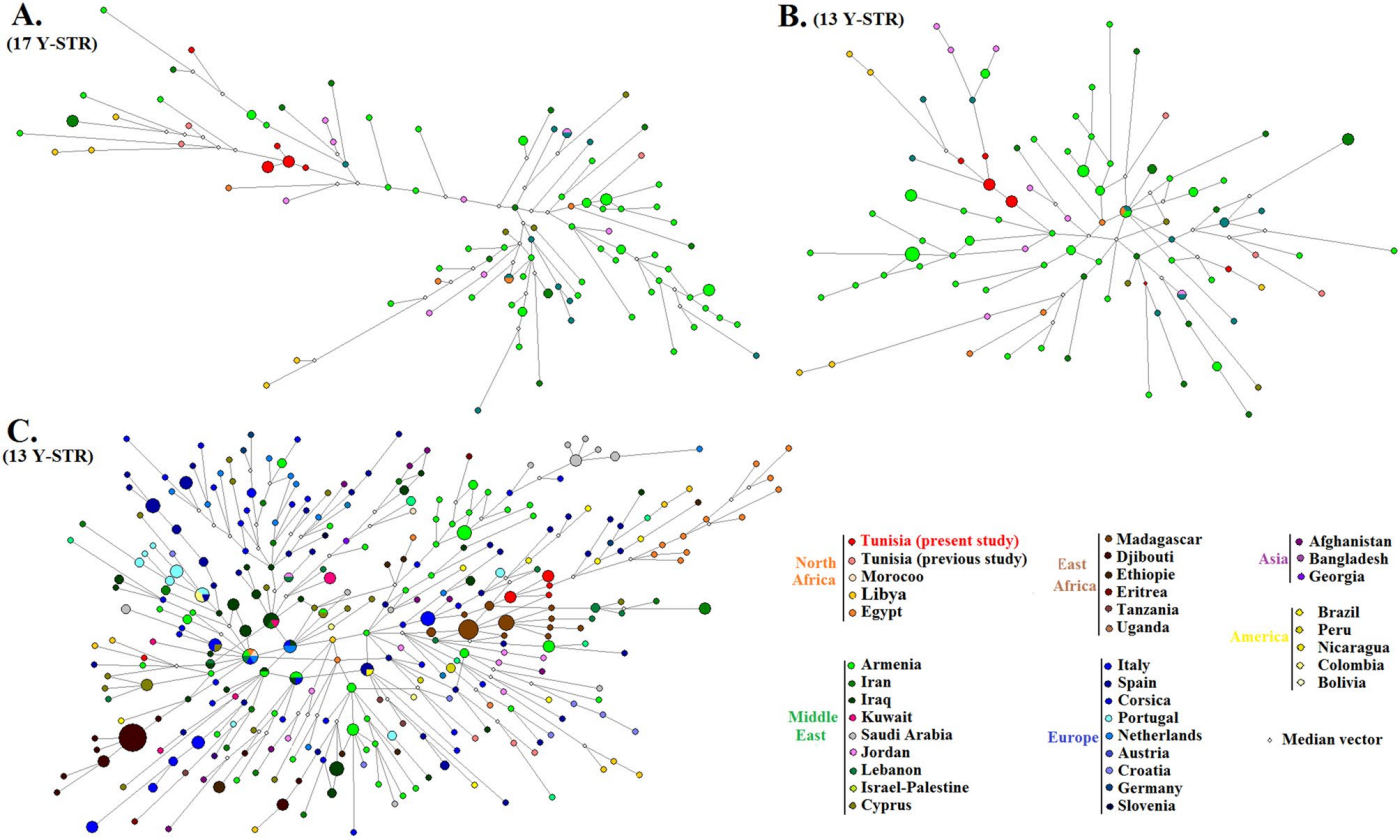
Median-joining networks of haplogroup T based on 17 Y-STR (**A**) and 13 Y-STR (**B**) in North African and Middle Eastearn samples; and in worldwide populations (**C**) using 13 Y-STR loci. The samples are color-coded according to the population they belong to (see Supporting Information Table S5) and the size of the circles is proportional to the frequency of a given Y-STR haplotype as the smallest circle is equal to one sample. The lengths of the connecting lines are proportional to the number of mutational steps separating two haplotypes.

To examine in depth this haplogroup in a broad scenario encompassing Tunisian and worldwide populations, a phylogeographic analysis of T lineages was undertaken, constructing three median joining networks: one based on a set of 17 Y-STR loci typed in 114 samples from North Africa and Middle East regions; other with the same samples but reducing the set of loci to 13 Y-STRs; and a third with the 13 Y-STRs used in the second network integrating 453 samples from worldwide populations (Fig. 3A–C, respectively; Supporting Information Table S5).

All networks illustrate the extensive divergence between haplotypes belonging to haplogroup T as well as the scarcity of shared haplotypes between populations and the faint geographic structure. The networks containing only North African and Middle Eastern individuals (Fig. 3A,B) show that when the number of Y-STR is augmented from 13 to 17 loci, some sub-clustering of T lineages emerges with the latter set, indicating that the resolution of SNPs here used to define T lineages was not enough to discriminate the heterogeneity that exist within this haplogroup, which is now well established (https://isogg.org/tree/).

The network with worldwide individuals (Fig. 3C), encompasses 322 different haplotypes, out of which only 11 were shared haplotypes (between individuals from different populations). Despite having been constructed with the small set of 13 Y-STRs, it reveals that the level of differentiation among haplotypes is especially high in those from the Middle Eastern, a finding that is consistent with the reported origin of T haplogroup in the Middle East^34^, after which it underwent a complex dispersal into Europe along with its spread in East and North Africa. But instead of a star-like structure (which could illuminate the source population where a primordial past expansion occurred), the network displayed a number of signals of evolutionary events leading to local diversification of lineages.

A subset of haplotypes from the Middle East presents several branches with many one-step neighbour haplotypes particularly from Armenia and North Iraq, which seems to be coherent with a presumed expansion of haplogroup T from the region of Fertile Crescent^50^. Connected to these haplotypes are many other from Iranian, Jordanian, Lebanese, Saudi, as well as from Turkish and Greek Cypriot profiles. Haplotypes from Israel-Palestine and Kuwait scattered throughout the network.

There are also various branches integrating haplotypes from Europe that are quite divergent between each other, indicating that the diversification of the T lineages introduced from the Middle East already occurred in loco in some European populations, namely from Italy, Spain and Portugal. Other European haplotypes (from Netherlands, Austria, Croatia, Germany and Slovakia) are dispersed throughout the network, as well are the few haplotypes from American and Asian populations which were randomly connected with both European and Middle Eastern representatives.

The network discloses in addition notable signatures of founder effects in two populations from East of Africa, illustrated by two large nodes, one of which only containing shared haplotypes from Djibouti (17 Somali who belong to the same clan family)^52^ and the other uniquely shared haplotypes from Madagascar (9 individuals). Although these two nodes encompass haplotypes quite divergent, both present core haplotypes considerably ramified.

The haplotypes from the studied Arab Tunisian populations (Kairouan and Wesletia) are connected to the Madagascar branch, to which are also related the Egyptian chromosomes, suggesting that all these haplotypes have a common source. Still, other haplotypes from Morocco, Egypt, Libya and Tunisians from Sousse are ubiquitously distributed across distinct branches from the network.

The TMRCA of Y-chromosome haplogroup T was 45.582 ± 5.162, in accordance with the range of values estimated by Hallast et al.^59^, while the TMRCA estimate for the branch including Arab Tunisian samples from Wesletia and Kairouan was 9.237 ± 6.450YBP.

### Interpolation analysis of Y-chromosome Middle Eastern haplogroup T

The interpolation map of the Middle Eastern haplogroup T in worldwide populations (Supporting Information Figure S2, Supporting Information Table S6) reveals that the geographical distribution of the T haplogroup is consistent with its assumed Eastern origin, as indicates its widely geographical area, with the highest frequencies in the Sasun population from Armenia (20.19%). Probably, T lineages dispersed first throughout Middle Eastern populations and then expanded into East Africa, where they occur at high frequencies in Djibouti (55.56%) and Madagascar (20.16%). In North Africa its distribution is rather irregular, but it is very common in Arabs Tunisians from Wesletia (17.4%). In Europe, the haplogroup is mainly disseminated in Southern regions, (Portugal 11.03%, Sicily 5.51% and Corsica 4.05%), likely reflecting trans-Mediterranean population contacts. From the scarce data available, it appears to be very low prevalent in Central and West Africa.

## Discussion

In this study, we have investigated the paternal diversity in Tunisian populations in order to add new insights on the complex demographic history that shaped current-day individuals from Tunisia and, more broadly, from North Africa.

One of the most important findings herein was the strong differentiation between the assumed Arabs from Kairouan and Wesletia, two very close cities in Tunisia (50 km away). Actually, whereas in the Arabs from Kairouan clearly prevailed a male genetic background associated to a Berber ancestry (high frequency of 73.47% of E-M81; position in the MDS plot in Fig. 2), in the Arabs from Wesletia was very high the component typically found in Middle Eastern populations (52.2% J-12f2a and 17.4% T-M70), largely surmounting the fraction connected with the North African substrate (21.7% of E-M81; position in the MDS plot in Fig. 2). This result reinforces the differentiation between these two populations previously documented through the analysis of the maternal diversity revealing that Wesletia exhibited a rate of Middle Eastern mtDNA lineages (28.1%) larger than Kairouan (12%)^38^.

At first glance, this is counterintuitive because Kairouan is recognised as having been an important Arabo-Muslim base in North Africa during five centuries (7th–11th), and even today it continues to be one of the holiest cities in Islam^35^. Thus, the question arises on whether the individuals from Kairouan might represent in fact arabized Berbers who have changed their original Berber surnames to Arab family names. This is not difficult to understand having in mind the social-cultural constrains that since long induced Berber people living in Kairouan to adopt family names associated to a higher status, and hence to mitigate a non-Arab descent when the city became the political and learning centre for a growing Arabo-Muslim elite in a region that experienced strong social polarization^36^. Furthermore, in 1056 all the zone where Kairouan locates was trashed by the invasion of Bedouin tribes (Banu Hilal and Banu Soleim) coming from the Arabian Peninsula in revenge for the Zirid dynasty that broke away from the Fatimid Empire^60^, an event that would be considered the major driver of the linguistic and cultural arabization of the indigenous Berber populations.

In turn, Wesletia and surrounding villages were the first localities in the area to be occupied by Arab Muslim troops from the beginning of the Arab conquest in 666. These rural cities reached the peak of their development until the 13th CE providing the neighbouring regions fruits, vegetables and good water quality^61^. Still today, several families from the region claim to have Middle Eastern origin.

Our sampling criterion relied on the self-provided patronymic information given by the voluntary participants in the study, which admittedly might be misleading on family ancestry as a consequence of the acculturation behaviour that was strongly rooted everywhere in Tunisia. However, since the criterion was applied both in Kairouan and Wesletia, the detection of very disparate amounts of Middle Eastern and Berber backgrounds in populations from the two regions might indeed reflect a higher influence of people from the Arabian Peninsula in Wesletia than in Kairouan.

The differences captured between Wesletia and Kairouan highlight the genetic heterogeneity of Tunisian populations, even sharing a common language and being located in close geographical proximity. That is also the case of the two Tunisian Andalusian populations Qalaat-El-Andalous and El Alia that are only 15 km away. In Qalaat-El-Andalous, two unique haplogroups E1b1b (89.5%) and J1 (10.5%) were found, while in El Alia 9 different haplogroups were observed encompassing almost equally frequent North African (44.19% of E1b1b1) and Middle Eastern representatives (overall 41.87%; J1, J2, G and T), largely exceeding the much less frequent European (6.97%; I2 and R1b), Sub-Saharan (4.65%; R1b1a2) and Asian lineages (2.32%; C2). Notwithstanding, there were also two Tunisian Andalusian populations where the Middle Eastern component dominates, as in Zaghouan (46.55%) and in El Alia (41.87%). Tunisian “Andalusians”, is the common description of those Tunisian populations that incorporated the Muslim people living in Al-Andalus who were expulsed from the Iberian Peninsula and entered the North of Tunisia at the beginning of the seventeenth century. That wave of migrants was essentially constituted by the descendants of the Muslims who entered and settled in Hispania following the Umayyad conquest of the territory (711–718 CE). It seems thus very likely that these newcomers to Tunisia had retained a strong Middle Eastern signature of their origin, which resisted even after admixture with populations mainly from Northern of Tunisia in which they were assimilated.

Considering Tunisian populations as a whole, the majority part of their paternal haplogroups are of autochthonous Berber origin (71.67%), which co-exists with others assumedly from the Middle East (18.35%) and to a lesser extent from Sub-Saharan Africa (5.2%), Europe (3.45%) and Asia (1.33%).

Most of Tunisian populations here analysed exhibited high levels of Y-STR diversity, reflecting the wealth of demographic histories underlying current-day populations. This finding is in agreement with previous studies using different types of markers that reported the highest genetic diversities in North African populations^17,37,38,43,62–65^. In the Tunisian population landscape, clearly conflicts the low values of diversity in the Arabs from Zriba and in the Tunisian Andalusians from Qalaat-El-Andalous. This can be explained because both are small sized populations that underwent remarkable drift effects^39,40^, as also applies to the Berber communities from Jradou, Bou-Omrane and Bou-Saad, among which drift effects and the endogamy promoted by cultural isolation must have interplayed to reduce diversity^12,13^.

Despite the remarkable differentiation between Tunisian populations, no clear factor of differentiation emerged when assuming the conventional assignment in Arabs, Andalusians, Berbers and Cosmopolitans, or when accounting for their geographical region of origin. This indicates that such kind of categorization or geography are irrelevant to explain substructure amongst Tunisian populations, which must thus rely in a complexity of interacting factors difficult to discern.

In this work, we further explored the male Eastern contribution to Tunisian populations which encompassed an average proportion of 18.35%. Importantly, in the Arabs from Wesletia that component attained 71.8%, which is the highest frequency among North African populations studied to date. The male lineages that better testify the influence of the Middle East, belong to haplogroup J-12f2a, which was carried by 52.2% males from Wesletia. In addition, in Wesletia another Middle Eastern haplogroup, T-M70, reached the frequency of 17.4%, a remarkable value once up to now the haplogroup had only been occasionally detected in North Africa, with the existing reports documenting its occurrence at rather low frequencies in Egypt and Libya. This prompted us to perform a detailed phylogeographic analysis of that haplogroup. According to Herrera et al.^50^, the T lineages originated in the Near East during the Paleolithic and were introduced in Armenia by migrations dating back to ∼12–13 kya, affording afterwards the time needed for considerable in situ diversification. The analyses undertaken in this study, pointed toward an ancient introduction of T lineages into North Africa, as illustrates the high level of molecular divergence among haplotypes. In the network of worldwide chromosomes, a branch is defined containing the T haplotypes from the Arab Tunisian population here studied, demonstrating a certain extent of local diversification (Fig. 3C.). Furthermore, that branch was close to the lineages from the Eastern African population of Madagascar. Thus, and assuming that T haplogroup was originated in Middle East, very likely it was introduced in Africa in the course of multiple waves of migration, one of which might have been through the Horn of Africa, which is seemingly consistent with the presence in Djibouti and Tanzania of sets of haplotypes quite divergent from others. In North Africa, one of the routes of introduction might have been across the Suez Isthmus from the Levant, as insinuates the cluster of T-lineages showing tight affinities in their STR backgrounds that integrates lineages from Arab Tunisians, Egyptians, individuals from the Middle-East and people from Madagascar.

The presence of these lineages in Madagascar justifies to briefly revisit the demographic history of the island that saw a rapid settlement in the last millennium. Whilst several lines of evidence indicate that the peopling of Madagascar had strong Indonesian and East-African influences, the Arab-Islamic impact cannot be neglected^45^. Many Muslims reached and settled Madagascar until around the fifteenth century, although their origin is still debated, with some advocating an arrival from Eastern Africa and others directly from the Middle East/Arabic Peninsula. In agreement with Capredon et al.^45^ the analyses here performed favours the second scenario, given the signs that the T lineages from Madagascar share a common origin with many from North Africa and Middle East.

The cluster that concentrates Arab Tunisian Y-chromosomes was estimated to be around 9000 ya old. This ancient age can be explained assuming two scenarios. First, it is reconciled with gene inflow from the Middle East since prehistoric times, probably going back to the Capsian civilization that flourished in North Africa around 8000 ya, which was a culture associated to the spread of agriculture into the region. Influences from the Middle East in the region of Wesletia left marks in the remains of prehistoric human occupation of the region, at least from Neolithic times on, in many refuges of Jebel Ousselat such as Ain Khanfous, Chendoube, Knefissa, Oued Grabech and Oued Bourrime, where abound rock paintings of domestic animals and hunting scenes^4,5^. Also the study of Fadhlaoui-Zid et al.^15^ sustained this scenario, estimating the coalescence age of J-M267 in Cosmopolitans from Sousse at 7.6 ± 5.2 kya; as well the recent phylogenetic analysis of Middle Eastern mtDNA lineages^38,66^ argues in favour of the eastern gene flow to North Africa during Neolithic periods as part of the Capsian civilization development. Notably for the mtDNA R0a and T1a haplotypes found in the Tunisian sample from Wesletia examined in this study, the TMRCAs were estimated around 9000 to 5000 ya^38^.

However, a second scenario cannot be discarded, in which the ancient age of the T lineages would not necessarily imply their ancient diversification in North Africa, but instead derives from a more recent introduction of lineages already well differentiated. This is compatible with the invasion of the Hilalian speaking tribes coming from the Arabian Peninsula during the eleventh century, among which T lineages might have been present since prehistoric times, resulting in substantial evolutionary diversification. The massive arrival of this people in North Africa may have been accompanied by gene flow enough to account, by chance, to the enrichment of the T lineages they possessed since prehistoric times.

These two scenarios are not mutually exclusive, and probably both contributed to the pattern of diversity of haplogroup T found in Tunisia that pointed towards the Neolithic as the period during which it began to accumulate heterogeneity.

In conclusion, the panorama obtained by the analysis of Y-chromosome polymorphisms confirms the mosaic structure of Tunisian population^67^. However, contrarily to the Tunisian maternal genetic pool that reportedly was mainly influenced by an European subtract (45.47%) in comparison with the much smoother Middle Eastern and North African mtDNA contributions (21.38% and 6.65%, respectively)^38,67^, the predominant component in the paternal pool in Tunisia was North African (71.67%) followed by an essentially Middle Eastern contribution (18.35%). This suggests that a sex-biased pattern of gene flow has modelled current day Tunisian populations, adding another level of complexity to their past that still needs to be further investigated. The phylogenetic analysis of haplogroup T revealed that it started to be introduced in North Africa in very remote times, probably through the Neolithic eastern genetic flow associated to the spread of agriculture, which was later reinforced by other population influxes into the region.

## Materials and methods

### Sampling and DNA extraction

Blood samples were collected from 95 unrelated healthy males originating from two regions of central Tunisia: 49 from Kairouan and 46 from Wesletia (Al-Waslatiyah). Only individuals providing patronymic evidence to belong to the Arab population were sampled. All subjects were volunteers who gave informed consent to participate in this study, which was performed with ethical approval of the local health authorities (the regional hospital of Kairouan and the local hospital of Wesletia) and the approval of ethics committee for research in life and health sciences (CER-SVS/04/2020) of the Higher Institute of Biotechnology of Monastir. DNA extraction procedure was based on the salting out protocol^68^ All study methods were carried out in accordance with the approved guidelines and regulations.

### Y-STR and Y-SNP genotyping

Samples were typed for 23 Y-STR loci (DYS19, DYS389I, DYS389II, DYS390, DYS391, DYS392, DYS393, DYS385ab, DYS437, DYS438, DYS439, DYS448, DYS456, DYS458, DYS635, GATAH4, DYS481, DYS533, DYS549, DYS570, DYS576, and DYS643) using the kit PowerPlex Y23 System (PPY23, Promega Corporation, Madison, WI). The amplified products were separated in an ABI 3130 Genetic Analyzer (Applied Biosystems) and analysed with GeneMapper v4.0 software (Applied Biosystems) using the supplied allelic ladders and internal size standard. In order to assign Y-chromosome haplogroups, the samples were genotyped with a set of three different multiplexes that included a total of 29 Y-SNPs: 10 Y-SNPs in Multiplex 1 + M13, 6 Y-SNPs in Multiplex 2 and 13 Y-SNPs Multiplex E^69,70^. We applied the haplogroup nomenclature proposed by http://www.phylotree.org/Y/.

A total of 132 Tunisian Andalusian samples originating from Testour (48), Slouguia (22), Qalaat-El-Andalous (19) and El Alia (43) were previously typed for 12 Y-STR^39,40^. We predicted their Y-haplogroups (which were not previously described) using Whit Atheys’ Haplogroup Predictor.

### Statistical analyses

Diversity parameters (number of different haplotypes, haplotype diversity and mean number of pairwise differences) were calculated using Arlequin software version 3.5.1.2^71^. The population genetic structure of the 18 Tunisian populations was assessed through several approaches: pairwise genetic distances of Slatkin’s (*R*_*ST*_) and analysis of molecular variance (AMOVA) pooling populations according to ethnical criteria, using the Arlequin software version 3.5.1.2^71^, along with Spatial Analysis of Molecular Variance (SAMOVA) performed with SAMOVA v1.0^72^. With the use of additional geographic information, SAMOVA maximizes the proportion of genetic variance due to differences among populations (*F*_*CT*_) for a given number of genetic clusters (K-value). Different numbers of groups (K = 2 to K = 7) were tested and then we considered the best grouping that yielding the highest *F*_*CT*_ value.

The genetic relationships between Tunisian populations and between Tunisian and other worldwide populations (North African, Sub-Saharan, European and Middle Eastern) were assessed by generating a Multidimensional Scaling (MDS) based on *R*_*ST*_ pairwise genetic distances for 12 Y-STR (DYS19, DYS389I, DYS389II, DYS390, DYS391, DYS392, DYS393, DYS385ab, DYS437, DYS438 and DYS439), using IBM SPSS Statistics version 19.0 software.

### Phylogenetic relationships

The median joining network of Y-chromosome haplogroup T was constructed with the Network software version 10.2 (http://www.fluxus-engineering.com/)^73,74^ using in the first step the set of 17 Y-STR loci (DYS19, DYS389I, DYS389II, DYS390, DYS391, DYS392, DYS393, DYS385ab, DYS437, DYS438, DYS439, DYS448, DYS456, DYS458, DYS635 and GATAH4) common to 114 samples from North Africa and Middle East regions which their haploroup classification to haplogroup T were based on Y-SNP data. Then, considering the same sampling, we generated a network using 13 Y-STR loci by omitting 4 Y-STR loci (the two multi-copy loci DYS385ab, DYS389II and DYS448). In a second step, we generated a network based on 13 Y-STR loci coincidentally typed in 453 individuals belonging to haplogroup T from 36 worldwide populations (4 North African, 6 East African, 9 Middle Eastern, 9 European, 3 Asian and 5 American), collected from the literature. For some populations, haplogroup classification, when not originally reported, was inferred from their Y-STR haplotypes using the online program Whit Atheys’ Haplogroup Predictor.

As for the inference of the TMRCA of T-M70 haplogroup, we used the mean pedigree mutation rate (6718) for the 13 Y-STRs in the case of a modal haplotype as a root, and as proposed by Hallast et al.^59^.

The T-M70 haplogroup frequencies obtained were used as the input data for a grid-based contour map, to study its geographic distribution. Surfer 8.0, mapping software from Golden Software, LLC, USA, was used for the frequency spatial distribution mapping. Kriging gridding method was adopted for the interpolation of geographical data https://www.goldensoftware.com/products/surfer. The assumed geographic location corresponded to the centre of the distribution area from where the individual samples were collected.

## Supplementary Information

Supplementary Legends.

Supplementary Figure S1.

Supplementary Figure S2.

Supplementary Tables.

## Data Availability

All data generated or analysed during this study are included in this published article and its supplementary information files.
