# Supplementary material for: Insights into the Middle Eastern paternal genetic pool in Tunisia: high prevalence of T-M70 haplogroup in an Arab population
Source: Sci Rep. 2021 Aug 3;11:15728. doi: 10.1038/s41598-021-95144-x (PMC8333252; doi:10.1038/s41598-021-95144-x)
Supplement: Supplementary file 1 — Supplementary Legends. [file 41598_2021_95144_MOESM1_ESM.docx]

**Supplemental Data files**

**Figure S1**. Multidimensional scaling plot based on *R_ST_* genetic distances between pairs of 18 Tunisian populations.

**Figure S2.** Interpolation map for Y-chromosome T haplogroup in worldwide populations. *(Coordinates, abbreviations of populations and frequencies of T haplogroup in Supporting Information Table S6).* Surfer 8.0, mapping software from Golden Software, LLC, USA, was used for the frequency spatial distribution mapping. Kriging gridding method was adopted for the interpolation of geographical data. <https://www.goldensoftware.com/products/surfer> *<a rel="license" href="http://creativecommons.org/licenses/by-nc-sa/4.0/"><img alt="Creative Commons License" style="border-width:0" src="https://i.creativecommons.org/l/by-nc-sa/4.0/88x31.png" /></a><br />This work is licensed under a <a rel="license" href="http://creativecommons.org/licenses/by-nc-sa/4.0/">Creative Commons Attribution-NonCommercial-ShareAlike 4.0 International License</a>.*

**Table S1.** 23 Y-STR haplotypes and Y-SNP haplgroup classification in Kairouan and Wesletia.

**Table S2.** Y-Chromosome haplogroups frequencies in Tunisian populations

**Table S3.** Pairwise F_ST_ genetic distances matrixes using Slatkin (R_ST_) (lower triangle) and corresponding *P*-values (upper triangle) between Tunisian populations.

**Table S4**. Populations analyzed for MDS.

**Table S5**. Y-chromosome STR haplotypes for Y-chromosome haplogroup T.

**Table S6**. Populations analyzed for contour map of Y-chromosome haplogroup T-M70.
