# Supplementary figures and images for: Insights into the Middle Eastern paternal genetic pool in Tunisia: high prevalence of T-M70 haplogroup in an Arab population
Source: Sci Rep. 2021 Aug 3;11:15728. doi: 10.1038/s41598-021-95144-x (PMC8333252; doi:10.1038/s41598-021-95144-x)

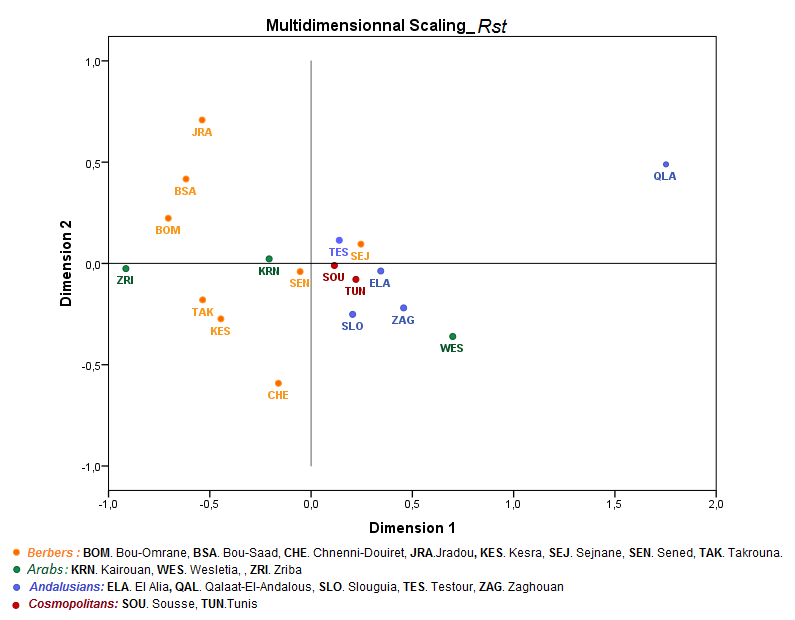

Supplement: Supplementary file 2 — Supplementary Figure S1. [file 41598_2021_95144_MOESM2_ESM.tif]

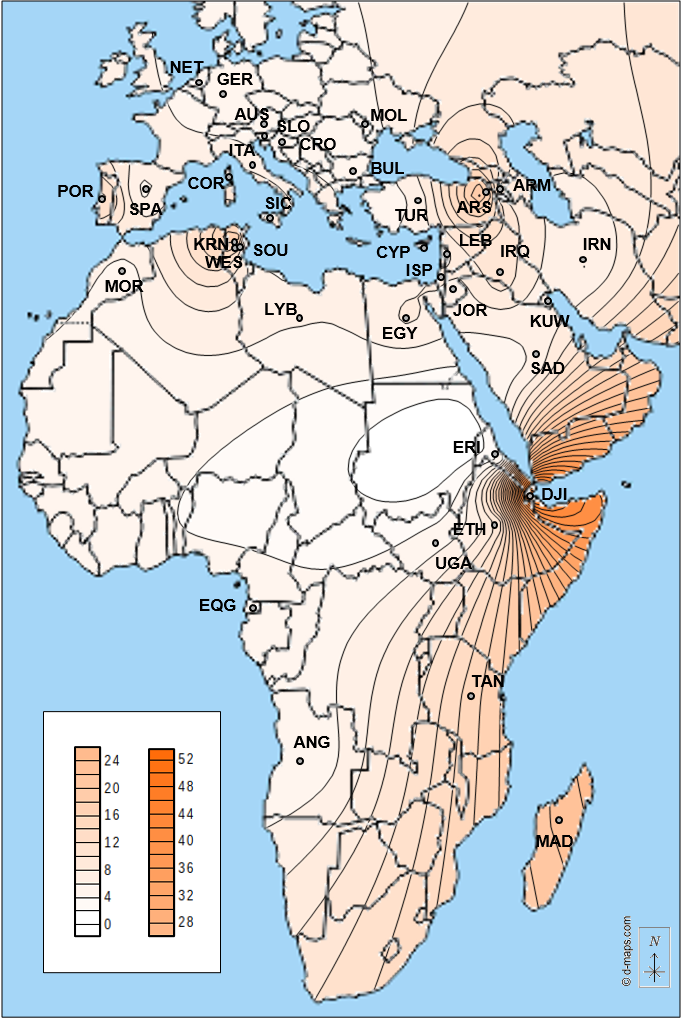

Supplement: Supplementary file 3 — Supplementary Figure S2. [file 41598_2021_95144_MOESM3_ESM.tif]
